# A Newly Integrated Model for Intestinal Cholesterol Absorption and Efflux Reappraises How Plant Sterol Intake Reduces Circulating Cholesterol Levels

**DOI:** 10.3390/nu11020310

**Published:** 2019-02-01

**Authors:** Takanari Nakano, Ikuo Inoue, Takayuki Murakoshi

**Affiliations:** 1 Department of Biochemistry, Faculty of Medicine, Saitama Medical University, Saitama 350-0495, Japan; t_mura@saitama-med.ac.jp; 2 Department of Diabetes and Endocrinology, Faculty of Medicine, Saitama Medical University, Saitama 350-0495, Japan; i1901018@saitama-med.ac.jp

**Keywords:** Adenosine triphosphate (ATP)-binding cassette G5/G8, brush border membrane, cholesterol absorption, fecal neutral sterol excretion, Niemann-Pick C1-like 1, plant sterol/stanol, trans-intestinal cholesterol efflux

## Abstract

Cholesterol homeostasis is maintained through a balance of de novo synthesis, intestinal absorption, and excretion from the gut. The small intestine contributes to cholesterol homeostasis by absorbing and excreting it, the latter of which is referred to as trans-intestinal cholesterol efflux (TICE). Because the excretion efficiency of endogenous cholesterol is inversely associated with the development of atherosclerosis, TICE provides an attractive therapeutic target. Thus, elucidation of the mechanism is warranted. We have shown that intestinal cholesterol absorption and TICE are inversely correlated in intestinal perfusion experiments in mice. In this review, we summarized 28 paired data sets for absorption efficiency and fecal neutral sterol excretion, a surrogate marker of TICE, obtained from 13 available publications in a figure, demonstrating the inverse correlation were nearly consistent with the assumption. We then offer a bidirectional flux model that accommodates absorption and TICE occurring in the same segment. In this model, the brush border membrane (BBM) of intestinal epithelial cells stands as the dividing ridge for cholesterol fluxes, making the opposite fluxes competitive and being coordinated by shared BBM-localized transporters, ATP-binding cassette G5/G8 and Niemann-Pick C1-like 1. Furthermore, the idea is applied to address how excess plant sterol/stanol (PS) intake reduces circulating cholesterol level, because the mechanism is still unclear. We propose that unabsorbable PS repeatedly shuttles between the BBM and lumen and promotes concomitant cholesterol efflux. Additionally, PSs, which are chemically analogous to cholesterol, may disturb the trafficking machineries that transport cholesterol to the cell interior.

## 1. Introduction

Cholesterol is an essential component of cell membranes and a source of the sterol derivatives [1]; however, metabolic dysregulation, such as hypercholesterolemia, is atherogenic and increases the risk of cardiovascular diseases (CVDs) [2], which account for ~31% of deaths globally (WHO, 2018) [3]. Cholesterol in the circulation originates from either the diet or endogenous synthesis in cells. To ameliorate abnormalities, drugs have been developed to inhibit cholesterol pathways. A meta-analysis showed that with pharmacological treatment that is initiated later in life, all-cause mortality was reduced by 10% per 1.0 mmol/L reduction in low-density lipoprotein cholesterol (LDL-C) [4]. This limited efficacy warrants further therapeutic approaches to attenuate the residual risk.

Cholesterol is excreted into feces as bile acids or in its original form. Approximately two-thirds of the latter is followed by bacterial 5β-hydrogenation into coprostanol and then coprostanone. This fecal excretion efficiency is inversely associated with carotid intima-media thickness, a measure of early atherosclerosis and the risk of CVD [5]. In addition to hepatobiliary cholesterol removal [6], the small intestine also eliminates endogenous cholesterol, the phenomenon for which was known as early as 1967 in humans [7] and is now referred to as trans-intestinal cholesterol efflux (TICE) [8]. TICE is considered to constitute approximately one-third of the total fecal neutral sterol (FNS) excretion in mice [9] and humans [10]. This pathway can be stimulated pharmacologically [11,12], and is thus a therapeutic target for CVD [13]. However, the mechanism of TICE is still unclear.

In this review, we provide a brief overview of the intestinal cholesterol absorption process, especially focusing on how cholesterol is taken up into the brush border membrane (BBM) (for details about intestinal cholesterol absorption, see reviews, e.g., [14,15,16,17]). Then, we will describe how cholesterol is handled in the intestinal mucosa with two cholesterol transporters, ATP-binding cassette G5/G8 (ABCG5/G8) and Niemann-Pick C1-like 1 (NPC1L1). By elucidating and integrating cholesterol dynamics, we will examine why TICE is mechanistically associated with cholesterol absorption. Our model is that the BBM of intestinal epithelial cells stands as the dividing ridge for cholesterol fluxes, meaning that cholesterol absorption and TICE are inversely correlated.

In the latter part of this review, we expand this idea to address how excess plant sterol/stanol (PS) intake inhibits cholesterol absorption and also stimulates TICE. We show how this food ingredient modulates cholesterol dynamics in the BBM and exerts favorable effects based on the bidirectional cholesterol flux model. 

## 2. Intestinal Cholesterol Absorption

### 2.1. An Overview of the Cholesterol Absorption Process

**Figure 1:**
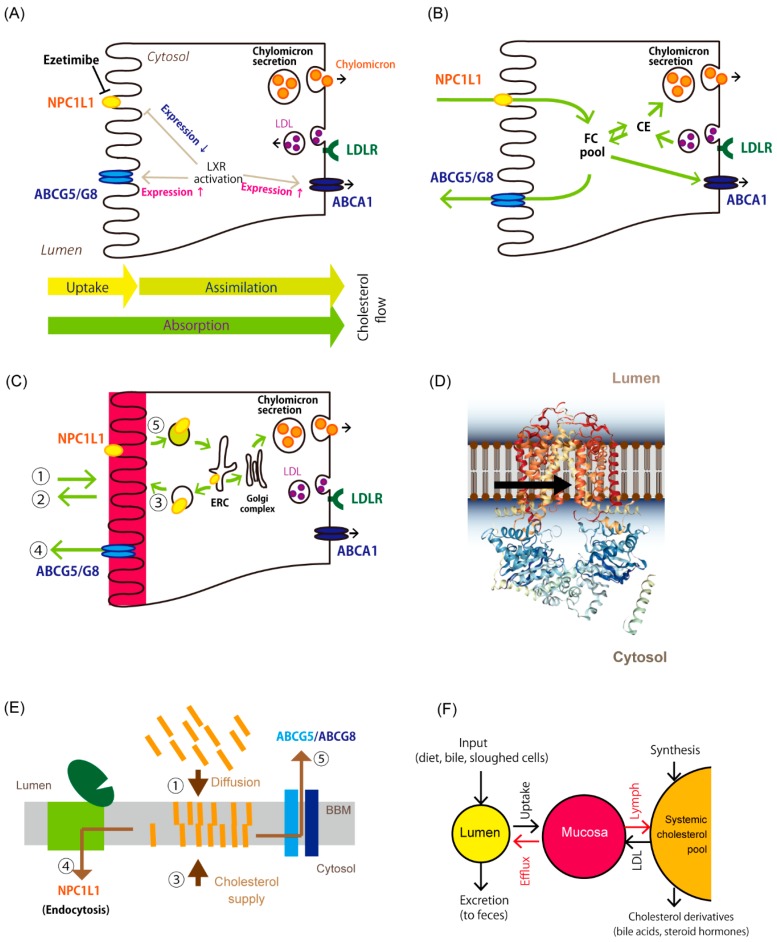
Overview of cholesterol trafficking in enterocytes. (**A**) Components associated with cholesterol absorption in enterocytes. Niemann-Pick C1-like 1 (NPC1L1) is the major cholesterol transporter. ATP-binding cassette (ABC) G5/G8 is an efflux transporter of sterols in the brush border membrane (BBM). Liver X receptor (LXR) modulates gene expression, the products of which are associated with the fluxes. Enterocytes take up cholesterol as low-density lipoprotein (LDL) via LDL receptor-mediated endocytosis from the basolateral side [19]. Cholesterol in enterocytes exists mainly as chylomicrons or very low-density lipoprotein (VLDL) [20]. These lipoproteins carry triacylglycerol predominantly and other lipids as well, but the figures do not show them for simplicity. Cholesterol is also transported into the serosal side to build high-density lipoprotein via ATP-binding cassette A1 (ABCA1). Arrows indicate up-regulation. Gene expression of NPC1L1 is attenuated by LXR activation. The terms of “uptake”, “assimilation”, and “absorption” are defined in the text. Ezetimibe is an NPC1L1 inhibitor. (**B**) A current image about how cholesterol transits in enterocytes. Cholesterol in the lumen passes through the membrane via NPC1L1. ABCG5/G8 effluxes free (nonesterified) cholesterol (FC) in the cytosolic area. CE, cholesteryl ester. (**C**) A newly proposed model for cholesterol movement in enterocytes. The BBM constitutes a large reservoir for free cholesterol (colored in red), which enters from and exists to the lumen in a diffusive manner (pathways 1 and 2). Cholesterol originating from de novo synthesis or the circulation is supplied to the BBM via vesicular transport (pathway 3). ABCG5/G8 effluxes cholesterol to maintain its abundance using energy from ATP hydrolysis (pathway 4). Cholesterol in the BBM moves to endocytic recycling compartments (ERC) via NPC1L1-mediated vesicular trafficking (pathway 5), then the Golgi complex, and packed into chylomicrons to be secreted into the circulation. (**D**) Topology of ABCG5/G8 and its cholesterol reception for transport. The resolved crystal structure of ABCG5/G8 showed that it is likely to obtain substrates from transmembrane site [21]. A three-dimensional image of ABCG5/G8 was obtained from the Orientations of Proteins in Membranes (OPM) database (http://opm.phar.umich.edu/protein.php?search=5do7). (**E**) ABCG5/G8 and NPC1L1 coordinate bidirectional cholesterol fluxes from the BBM. The BBM receives cholesterol from internal and external sources. NPC1L1 is endocytosed with membrane cholesterol in a regulated manner [22,23]. ABCG5/G8 effluxes excess sterols from the BBM. (**F**) A simplified open compartment model for cholesterol pools in the body (for details, see [5,20,24]). Circles indicate cholesterol pools in the body: *Lumen*, cholesterol in the intestinal tract; *Mucosa* reserves predominant cholesterol in the intestinal BBM. Arrows in red show cholesterol fluxes mediated by ABCG5/G8 and NPC1L1 for efflux and the basolateral cholesterol secretion (Figure 1E), respectively. Epithelial cell sloughing/shedding and nontransporter-mediated efflux (Figure 1C, pathway 2) also mediate mucosa-to-lumen cholesterol transition. The functionality of these transporters plays a crucial role in the net fluxes of cholesterol from the mucosa.

The small intestine is the site where cholesterol absorption takes place. “Absorption” of cholesterol includes multiple steps from the uptake and metabolism of cholesterol to its transfer into the circulation via the thoracic duct. We have divided “absorption” into two processes: “uptake” of cholesterol, which refers to the entry of cholesterol into intestinal epithelial cells, and “assimilation” of cholesterol, which indicates the transfer of cholesterol from the cell interior to the lymph via the basolateral membrane (Figure 1A). In the latter process, cholesterol esterification, packaging into lipoproteins, and exocytosis are included. Dietary lipids, such as triacylglycerol, phospholipids, cholesterol and their derivatives, fat-soluble vitamins, noncholesterol sterols are also absorbed via the same pathway. Figure 1B shows a conventional cholesterol flux model in enterocytes, which can be seen in previous reviews, e.g., [16,18].

### 2.2. Passive Diffusion Mediates Intestinal Cholesterol Uptake

In addition to diet, bile and sloughed epithelial cells from the intestinal wall also supply cholesterol within the intestinal lumen, reaching 2–3 g per day in total [15]. Cholesterol solubilized into lipid micelles in the lumen penetrates the unstirred water layer of the intestinal wall and reaches the BBM, the primary cholesterol reservoir in the intestine. Unesterified cholesterol constitutes about one-third of BBM lipids (Cholesterol:phospholipid = 1:2) [25], in which cholesterol is densely packed as microvilli with a vast epithelial surface area. Experiments in vivo showed that the uptake process is mediated by passive diffusion [26,27,28] (Figure 1C), which is the amount uptake is increased in relation to the concentration in the lumen. 

Passive diffusion is likely to occur considering the physico-chemical nature of the interaction between hydrophobic compounds, such as cholesterol, and lipid bilayer membranes [29]. Compassi et al. [30] showed that the cholesterol incorporation capacity of the BBM decreased by protease treatment in vitro, suggesting that it was a protein-mediated process. However, proteins are the predominant constituent of prepared BBM vesicles, accounting for two-thirds of the weight [31]. Therefore, protease treatment could tear apart BBM vesicles and reduce the retention capacity for sterols. Moreover, because many of the proteins in the BBM constitute cholesterol-rich microdomains; thus, disturbance can also impair the retention capacity. Furthermore, there have been no protein molecules identified that affect uptake. Cholesterol uptake by intestinal BBM vesicles from mice was unaffected by the deletion of genes associated with intestinal cholesterol absorption (*NPC1L1*, *SCARB1*, and *CD36*) [32,33]. These observations suggest that uptake itself is concentration dependent and not protein mediated.

### 2.3. NPC1L1 Is a Major Gatekeeper for Cholesterol Assimilation in Enterocytes

Cholesterol taken up by the BBM is transferred to the endoplasmic reticulum (ER) to be esterified. The endocytic pathway organizes mobilization from the BBM to the ER [34]. NPC1L1 is a polytopic transmembrane protein and is the key molecule for this process [35]. Disruption of the coding gene reduces cholesterol absorption by half in mice [35]. This transporter resides in the intestinal BBM and internalizes upon exposure to cholesterol on the apical membrane in the porcine jejunum [22], which was inhibited by ezetimibe [23]. Inhibition of the internalization by ezetimibe was recapitulated in vitro [36], although Johnson et al. found no such effect [37]. The inconsistency could be due to the difference of assay conditions, especially cholesterol supply from the medium [36], which dramatically affects the protein localization in the cells. The endocytic machineries and mechanisms have been elucidated mainly in hepatocytes [38,39]. A similar system may function in the small intestine. 

The N-terminal domain of NPC1L1 has a cholesterol-binding pocket [40]. Additionally, the protein contains a sterol-sensing domain to accommodate one molecule of cholesterol [41]. These are likely to act to sense and initiate clathrin-mediated endocytosis. NPC1L1 resides in cholesterol-rich microdomains; thus, endocytosis can drag in several cholesterol molecules at once (Figure 1C,E) [38]. Indeed, NPC1L1-mediated endocytosis is also associated with the absorption of dietary fat-soluble vitamins E and K [42,43,44,45,46], probably by non-specific incorporation of membrane fragments together with NPC1L1.

Although dietary lipid triacylglycerol is absorbed almost completely, only approximately half of cholesterol in the lumen is absorbed, and this absorption exhibits large inter-individual variability [47]. Furthermore, the absorption is a slow process. When radiolabeled cholesterol was given in the diet, appearance in the circulation peaked 24–48 h after ingestion, whereas triacylglycerol levels peaked after 2 h [48,49]. Chylomicron secretion with a subsequent meal ingestion stimulates the appearance of labeled cholesterol in the circulation, indicating that cholesterol is retained and pooled in the intestinal epithelia [48]. 

## 3. The Small Intestine Excretes Endogenous Cholesterol

### 3.1. Trans-Intestinal Cholesterol Efflux

In 1959, Cheng et al. [50] observed diet-unattributable FNS excretion in patients with complete biliary obstruction. In 1967, Simmonds et al. [7] reported direct evidence for the phenomenon through intestinal perfusion studies in humans. Intestinal cell sloughing or shedding was given as an explanation. In 2007, van der Velde et al. [9] showed that BBM-to-lumen cholesterol efflux or secretion occurs physiologically in mice. Later, two independent research groups demonstrated this mechanism in humans. Jakulj et al. [10] calculated the contribution of TICE in FNS excretion in basal and ezetimibe-stimulated conditions in healthy volunteers. Moreau et al. [51] confirmed that excretion of plasma D7-cholesterol into feces occurred in 2 patients with complete common bile duct obstruction. 

In this review, we specify that TICE eliminates cholesterol from the body via the small intestinal wall by transporters or by being pulled out nonspecifically in lipid soluble conditions with amphipathic molecules, such as bile acids [9,52]. Cholesterol elimination by epithelial cell sloughing or shedding, i.e., “passive” TICE [51] should be included as a non-regulatory fraction of TICE. 

Although cholesterol is absorbed and synthesized in the body to supply cells, endogenous excess cholesterol is eliminated from the circulation via the gut, not only for balancing overall cholesterol abundance but also for physiological cholesterol turnover. TICE constitutes up to 70% of FNS excretion in mice [8]. With indirect calculations and compensation, human TICE is estimated to be approximately 35% of that in basal conditions [13]. Because the contribution of TICE to FNS excretion was based on indirect measurements, it may differ among analytical settings. Even though, these show that TICE is at least one of the two reverse cholesterol transport pathways that excrete large amounts of endogenous cholesterol from the body and can be stimulated for treatment or prevention.

**Figure 2:**
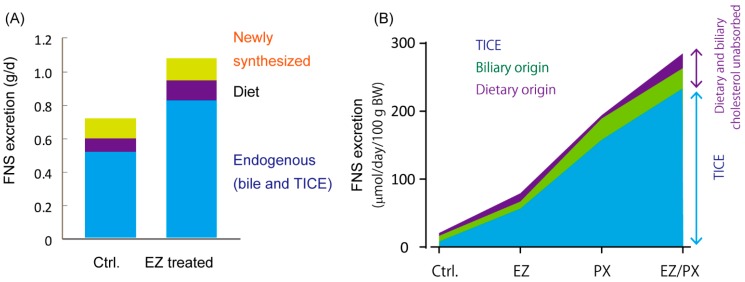
Stimulation of fecal neutral sterol (FNS) excretion represents an increase in trans-intestinal cholesterol efflux (TICE). (**A**) Ezetimibe (EZ)-stimulated FNS excretion results from an increase in endogenous cholesterol secretion into the gut lumen in humans, as determined by quantitative analysis with stable cholesterol isotopes (Data are obtained from Reference [56]). These findings indicate that increased FNS excretion is not attributable to the fraction of cholesterol left unabsorbed. (**B**) TICE dominates in the increase of FNS excretion in mice. Calculation of TICE in mice treated with EZ, PX20626 (PX), or both shows that the increase in FNS excretion originates from stimulated TICE (Data are obtained from Reference [11]). PX20606, a farnesoid X receptor agonist.

Notably, deletion of the gene encoding *NPC1L1* increased FNS excretion [53]. Studies conducted in humans and mice have shown that the potent NPC1L1 inhibitor ezetimibe stimulates TICE by 45% in direct TICE measurements in mice [54], by approximately 3–4-fold in mice in FNS excretion [10,11,12], and by 52% [55] and 67% [56] in humans in FNS excretion. With the treatments, unabsorbed dietary and biliary cholesterol contributed to increased FNS excretion only partly, whereas FNS excretion originating from endogenous cholesterol constituted the major part [56] (Figure 2A). Quantitative analyses with stable isotopes in mice showed that increased FNS excretion was attributable to augmented TICE [11] (Figure 2B). On the other hand, there were only marginal changes in the biliary cholesterol secretion rate. Indeed, NPC1L1 is not expressed in the liver of mice [35], excluding a hepatic contribution to the increase.

### 3.2. ATP-Binding Cassette G5/G8 Heterodimer Plays a Major Role in TICE

The heterodimer composed of ABCG5 and ABCG8 is a member of the ABC transporters. It is located in the BBM and is responsible for the efflux of cholesterol and noncholesterol sterols. In addition to a counteracting action against cholesterol absorption [57,58], it has been revealed that ABCG5/G8 provides a major efflux pathway. Disruption of ABCG5/G8 genes abolished TICE partly when it was stimulated, although the disruption did not affect basal TICE, indicating that ABCG5/G8 is required for efficient and enhanced TICE [11,54]. TICE may also occur with other transporters, such as ABCB1a/b [59]. 

Lee et al. [21] resolved the crystal structure of ABCG5/G8 in lipid bilayers, revealing that cholesterol ‘vestibules’ open to the inner leaflet of the plasma membrane (Figure 1D), indicating that it is the entry path of sterols and the transporter captures cholesterol from the BBM [60]. 

### 3.3. Cholesterol Absorption Inhibition by Liver X Receptor Agonism also Stimulates TICE

A liver X receptor (LXR) agonist T0901317, which up-regulates elimination-prone gene expression, e.g., ABCG5, ABCG8, and ABCA1, and down-regulates NPC1L1 in the small intestine [61,62] (Figure 1A), not only reduced intestinal cholesterol absorption but also increased FNS excretion in an ABCG5/G8-dependent manner [63,64]. The increase in FNS excretion with another LXR agonist, GW3965, was not affected in *MDR2*^−/−^ mice, in which biliary cholesterol excretion is lacking, again excluding a biliary contribution to the increase [64]. These findings also support the involvement of the two transporters in the coordination of cholesterol flux in the BBM.

## 4. A Newly Integrated Model for Cholesterol Bidirectional Fluxes in the Small Intestine

**Figure 3:**
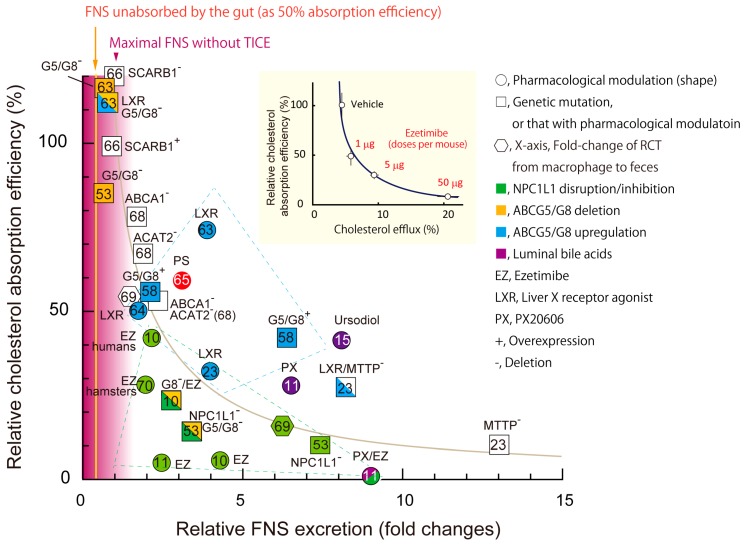
The relationship between intestinal cholesterol absorption and fecal neutral sterol (FNS) excretion. *Inset*, the figure was originally published in Nakano, T., et al. *PLoS ONE* 2016; *11(3)*: e0152207 [54]. The original titles for the X- and Y-axis were “% of DPM appearing in the lumen/DPM in intestinal segment (Efflux efficiency)” and “Relative lumen-to-circulation ^3^H-cholesterol transit (% vs. vehicle)”, respectively. The titles were changed to simplify the figure. An inverse relationship between absorption and TICE was hypothesized in the paper. The numbers (micrograms) in the figure indicate the dosages of ezetimibe given to mice. All 28 data sets presented in the figure were obtained from 13 published papers. Cholesterol absorption efficiency (%) indicates relative ratios compared with the respective control groups, for example, wild-types for transgenic mice and vehicle administration for pharmacological treatments. *Circles*, chemical treatments; *squares*, transgenic mice or those with chemical treatments; *hexagonal shapes*, macrophage-to-feces reverse cholesterol transport. EZ, ezetimibe, LXR, liver X receptor agonist; PX, a farnesoid X receptor agonist PX20606. +, mice with overexpression of the indicated gene(s); −, mice with deletion of the indicated gene(s). *Green*, NPC1L1 was disrupted by genetic deletion or by EZ treatment; *yellow*, genes for ABCG8 (G8) or both ABCG5 and G8 (G5/G8) were deleted; *blue*, ABCG5/G8 expression levels were activated by a LXR agonist or genetic modification(s). ACAT2, acyl-CoA acyltransferase 2; ABCA1, ATP-binding cassette A1; MTTP, microsomal triglyceride transfer protein; SCARB1, scavenger-receptor B1. Mice were used as the model unless mentioned otherwise in the plots. Letters in the symbols indicate the references the data were obtained from. The areas shown as blue and green dotted lines indicate experiments with LXR agonists or ABCG5/G8 overexpression, and those with EZ or NPC1L1 deletion, respectively. Yellow vertical line indicates basal FNS excretion originating from unabsorbed cholesterol as 50% absorption efficiency. Purple gradation indicates the approximate maximal FNS excretion originated from unabsorbed cholesterol [52,65,66,67,68,69,70].

We reported previously that BBM-to-lumen cholesterol efflux, i.e., TICE, is inversely correlated with intestinal cholesterol absorption efficiency [54] (Figure 3, inset). The data shown in Figure 2 indicate that increased FNS excretion mainly originates with TICE; thus, it can be assumed that increased FNS excretion was TICE. Figure 3 shows an analysis of changes in FNS excretion and intestinal cholesterol absorption (28 pairs) obtained from 13 published papers to date, demonstrating and supporting the inverse correlation between these two parameters. So far, it has been depicted that ABCG5/G8 and NPC1L1 independently translocate cholesterol [16,18] by accepting cholesterol from the cytosol and unstirred water layer, respectively (Figure 1B). With recent evidence, we reconsidered the roles of the two transporters, as described below and depicted in Figure 1C,E.

The BBM reserves mucosal cholesterol predominantly (Figure 1C) [71]. Figure 1F shows a simplified open compartment model for cholesterol pools and fluxes in the body [71]. The intestinal tract, especially the upper small intestine, is the second greatest organ to synthesize cholesterol [72,73]. In addition to cholesterol absorbed from the lumen, newly synthesized cholesterol remains in the villi, with a slow translocation to either the lymph [13] or the lumen (approximately 4% per hour [24]), providing a large pool of cholesterol in the mucosa [73,74] (Figure 1F, Mucosa). 

ABCG5/G8 and NPC1L1 probably accept mucosal cholesterol and transport to it in opposite directions (Figure 1E,F, red arrows; see Section 2.3 and Section 3.2). Thus, the BBM itself can function as the dividing ridge for cholesterol flux, and the transporter does not necessarily receive cholesterol from the aqueous cytosol (Figure 1B). The lymph flow determines the amount of cholesterol to move from the pool into the circulation. Meal intakes, especially fatty diets, augment the flow; thus, cholesterol absorption is enhanced accordingly [74,75]. Given that the cholesterol pool in the mucosa is maintained to be constant, TICE can be promoted when the cholesterol absorption is limited.

## 5. Plant Sterols Modulate Cholesterol Flux in the BBM

### 5.1. Plant Sterols and Their LDL-C Lowering Effect

Sterols are present in all eukaryotes. Land plants contain noncholesterol sterol analogs, PSs, which are chemically analogous to cholesterol, differing only in their side chain length [76]. The differences make them more hydrophobic than cholesterol [77]. Daily PS intake ranged from 200 to 400 mg in The EPIC-Norfolk cohort study [78]. A PS-enriched diet has been confirmed to lower LDL-C levels in many clinical studies [79,80]; this effect had already been shown in the 1950s in experimental animals [81,82]. The LDL-C lowering effect continues to increase up to intakes of approximately 3 g per day to an average effect of 12% in subjects [79,83]. This favorable effect has been attributed to the inhibition of intestinal cholesterol absorption by PSs with unknown mechanisms [84]. In this section, we hypothesize a novel mechanism for the effects of PSs based on the bidirectional flux model described above.

### 5.2. PSs as Modifiers of Cholesterol Flux in the Mucosa

Intake of PS stimulates FNS excretion by increasing TICE in mice [54,65]. Even with an extensive and long research history for PS-mediated LDL-C lowering, no study has addressed this notable effect except for that by Brufau et al. [65]. They raised PS content in the diet up to 8%, thereby observing a gradual increase in cholesterol absorption inhibition and 85% maximum reduction. On the other hand, 1% PS diet increased FNS excretion by more than 3-fold, but there was no such dose-dependent effect in it with a 1–8% PS diet, indicating a mechanistic limitation or saturation of TICE stimulation by PSs.

Subjects who carry a heterozygous inactivating mutation of NPC1L1 had much lower CVD risk even with a weak reduction of LDL-C [85]. In such conditions, TICE should be up-regulated (Figure 3). The increased FNS excretion should promote the renewal of circulating cholesterol-rich lipoproteins, especially LDL, throughout life [86,87]. LDL undergoes a variety of modifications in the body [88,89]. The modified products potently induce atherosclerosis [90]. Accordingly, the accelerated renewal or clearance of atherogenic lipoproteins may play a role in the favorable effect. 

The abovementioned inconsistency in the efficacies between the two parameters with PS-supplemented feed in mice [65] suggests a possibility that PS-supplemented diet stimulates FNS excretion at a low dosage with no apparent LDL-C lowering effect. If so, not only PS-enriched foods [91,92] but also just encouraging the consumption of PS-rich ingredients, such as nuts, seeds, vegetable oils, could be comparable to the partially NPC1L1-inactive phenotypes [85]. 

### 5.3. PS Transition to the BBM

**Figure 4:**
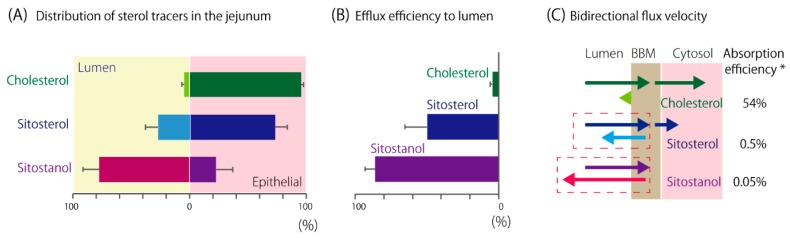
(**A**) Tracer distribution in the murine jejunum 3 h after oral infusion. Tracers in the luminal flush and in the intestinal segment are shown in percent of the total. Data were obtained from the studies conducted in Reference [54] and were reanalyzed. Assay replication: cholesterol, *n* = 10; sitosterol, *n* = 6, sitostanol, *n* = 11. (**B**) Percentages of effluxed tracers from the mucosa for 1 h during cannulated jejunal perfusion. Assay replication: cholesterol, *n* = 10; sitosterol, *n* = 6; sitostanol, *n* = 6. Similar tendency was also reported in mice by an independent research group [94]. (**C**) The diagram shows cholesterol or PS fluxes at the BBM. The lengths of the arrows indicate the supposed flux velocity. *Dashed boxes* indicate where PS-shuttling takes place. Data are shown as mean and standard deviation. *Absorption efficiency in humans was obtained from [95] for cholesterol and [96] for PSs.

Cholesterol and PSs have similar physicochemical natures, thus it is likely that they behave alike during movement between the unstirred water layer at the luminal surface and the lipid bilayer of the BBM [93]. Indeed, cholesterol and PSs were incorporated into the BBM to almost the same extent, when the incorporations were measured at an initial period after exposure in mice [30,94]. However, the measurement of cholesterol or PS tracer distribution in the murine jejunum 3 h after the oral infusion showed that higher hydrophobicity made the tracers accumulate in the lumen [54] (Figure 4A). In total, 77% of sitostanol tracer was recovered from the lumen, whereas only 4% of cholesterol was similarly recovered. We chased the efflux of tracers from the mucosa for 1 h and found that PSs are effluxed to the lumen from the tissue rapidly according to their hydrophobicity [54] (Figure 4B), consistent with the results by Igel et al. [94]. Efflux of sitostanol and sitosterol were 19-fold and 11-fold, respectively, greater than that of cholesterol. Accordingly, PSs that are effluxed into the lumen will be taken up again, which should result in repeated shuttling between the lumen and the BBM in the small intestinal tract (Figure 4C). This shuttling would promote concomitant cholesterol efflux by ABCG5/G8 (Figure 1C, pathway 3) and non-transporter-mediated efflux (Figure 1C, pathway 2). Thus, we think that PSs compete with the cholesterol absorption process both in the lumen and mucosa. 

### 5.4. Possible Sites Where PSs Compete with the Absorption Process of Cholesterol

**Figure 5:**
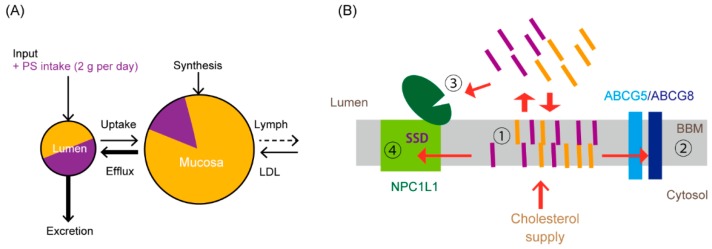
(**A**) Changes in cholesterol flux with plant sterol/stanol (PS) intake are shown in a simplified open compartment model in Figure 1F. Two grams of PS per day, which is recommended to reduce circulating LDL-C, could predominate in luminal sterols and is incorporated into the BBM (Mucosa). *Lumen* indicates the ratio of cholesterol to PS as 1:1, because approximately 2 g of cholesterol is supplied to the intestinal lumen per day in humans [15]. PS stimulates efflux, thus increasing total fecal neutral sterol excretion. These increased pathways are shown in bold arrows. PS in the mucosa and the lumen is indicated in purple as an image. (**B**) Possible effective sites of PSs for cholesterol absorption inhibition. The possible sites are indicated as ① to ④ in the figure. ①, cholesterol (yellow bars) and PSs (purple bars) are taken up by the BBM, where the sterol capacity is limited. ②, ABCG5/G8 accepts both substrates [98]. ABCG5/G8 might be activated by PSs [54,97,99,100] and efflux more cholesterol concomitantly. ③, PSs might dilute cholesterol at the surface of the mucosa, reducing the chance to access to the binding site in the N-terminal domain of NPC1L1. ④, PSs can be mixed with cholesterol in the BBM, reducing the abundance of cholesterol in it and preventing the sterol-sensing domain (SSD) of NPC1L1 from sensing an increase in cholesterol. PSs may also compete with cholesterol for the binding site of the SSD.

Cholesterol and PSs are both incorporated into the BBM, but the capacity of the BBM to accommodate sterols is limited, possibly resulting in an induction of sterol efflux from the BBM to the lumen. We show a modified compartment model of Figure 1F in an assumption that 2 g of PS is taken (Figure 5A). In summary, our hypothesis is as follows: PS abundance in the lumen reaches about half of the sterol/stanol content. The PS in the lumen induces PS-shuttling at the BBM (Figure 4C) and accelerates “trans-intestinal sterol efflux” (TISE), including cholesterol efflux (Figure 5A, bold arrow). This makes an outward flux and attenuates mucosa-to-lymph cholesterol flux (Figure 5A, dotted arrow). Thus, the BBM is a plausible site where PSs and cholesterol complete (Figure 5B, site number 1). We showed that PS perfusion in intestinal segments increased cholesterol efflux from the epithelia [97]. On the contrary, such an effect was not observed when the same amount of cholesterol was perfused. These findings support the hypothesis that luminal PSs can stimulate the efflux of cholesterol in the BBM.

Driven by ATP hydrolysis, ABCG5/G8 effluxes sterols back into the lumen. Interactions with bile salts can increase the hydrolyzing activity [99,100]. Rapid efflux of PSs by ABCG5/G8 implies a possibility that PSs stimulate ATP hydrolysis by ABCG5/G8 [54,94,97]. Thus, an increase in ABCG5/G8 activity in the presence of PSs might promote cholesterol efflux concomitantly (Figure 5B, site number 2). However, comparable PS-mediated fractional cholesterol absorption inhibition was observed even in mice lacking ABCG5/G8 [65,101], indicating that ABCG5/G8-mediated cholesterol efflux is not necessary [65]. Alternatively, cholesterol in the BBM can be effluxed into the lumen by a diffusion manner (Figure 1F).

NPC1L1-involved endocytosis, a process to facilitate cholesterol absorption, takes place when excess cholesterol is given to the lumen [22,23]. This means that excess cholesterol is sensed by NPC1L1 itself or NPC1L1-containing complexes. As analogous compounds to cholesterol, the presence of PSs may impair this sensing, leading to the attenuation of endocytic processes, although there are no available data on this possibility at present. NPC1L1 has a cholesterol binding pocket in its N-terminal domain (Figure 5B, site number 3). The binding site is relatively cholesterol specific and was not disturbed by adding sitosterol [40]; therefore, it is not likely to be a competitive site. The sterol-sensing domain (Figure 5B, site number 4) is also a possible competitive site, which needs to be elucidated in future studies. 

### 5.5. The Micellar Solubilization Hypothesis for PS-Mediated Cholesterol Absorption Inhibition

Several ideas have been postulated for the mechanism of PS-mediated cholesterol absorption inhibition [82]. Solubilization of sterols in the lumen is an indispensable process for the absorption, and sitosterol reduces cholesterol solubilization in mixed micelles [93,102,103]. Ikeda et al. showed that the presence of sitosterol limited the cholesterol transfer efficiency from micelles to the BBM in a series of in vitro and in vivo experiments [84,104]. 

Even with approximately 30–40% limited solubility in mixed micelles; however, the small intestine seemed to have a vast capacity to take up several hundred mg of cholesterol in a meal. Furthermore, it can take a couple of days to transit unabsorbed cholesterol through the intestinal tract [95]. Thus, the small intestine could compensate for the limited cholesterol availability from mixed micelles in the presence of PS. The micellar solubilization hypothesis presupposes the presence of PS in the lumen, or ingestion of cholesterol together with PSs to exert the effect. On the other hand, even when a PS-supplemented meal was taken once a day, a similar LDL-C lowering effect was obtained compared with the same amount of PS (for example, 2.5 g in reference [105]) taken three times a day at meals [91,105,106], implying that the presence of PS in the lumen is not necessarily required for the effect. Finally, we showed that TICE counteracts cholesterol absorption (Figure 3). PSs stimulate TICE [65]; thus, this augmentation of efflux from the BBM should be involved in the effect.

### 5.6. Association of PS Intake with LXR Activation

LXR mediates efflux-prone gene expression (Figure 1A). In differentiated Caco-2 cells, PSs activated this transcription factor, suggesting that PSs acting as ligands [107]. On the other hand, little effect on LXR-mediated gene expression was observed in the small intestine of PS-fed mice [108]. Moreover, the lack of ABCG5/G8, a target of LXR, did not attenuate PS-mediated cholesterol absorption inhibition [101] and only partly for increased FNS excretion [65]. These findings suggest that a transporter-independent pathway plays a role and can compensate the lack of the efflux transporter.

## 6. Trans-Intestinal Sterol Efflux

### 6.1. TISE 

We have shown that NPC1L1 and ABCG5/G8 play key roles in TICE. The two transporters transport cholesterol and noncholesterol sterols. The system thus provides the BBM with elimination of non-nutritious sterols/stanols and selective nutritious cholesterol uptake and can, therefore, be TISE. “Sterols” includes cholesterol, cholestanol, and their derivatives, and noncholesterol sterols/stanols. Thus, TICE is a part of TISE. TISE is inversely associated with sterol/stanol absorption principally. Indeed, circulating sitosterol and sitostanol have been used as positive cholesterol absorption makers [109]. 

### 6.2. Association of Circulating PS Levels with the Newly Integrated Model for Intestinal Sterol Absorption and Efflux

Metabolic disturbances, especially with dysregulated insulin signaling, in the small intestine induce exaggerated sterol/stanol absorption. This alteration can be defined by the consistent molecular signatures: decreased expression levels of *ABCG5/G8* and increased expression of *NPC1L1* in humans and rodents with metabolic abnormalities [110,111,112,113,114,115,116,117,118,119,120,121]. Therefore, TISE is weakened in such conditions. Patients with CVDs often have higher PS levels in the blood [122,123]. Accordingly, metabolic abnormality in such patients probably contributes to the increase [124]. Additionally, the genetic background of an individual can affect the absorption efficiency and levels [125]. Controversy exists whether circulating PS is a risk of CVD or not [122,126], but spillover, concomitant effect, or both need to be considered.

## 7. Concluding Remarks

In this review, we proposed a model where the BBM stands as the dividing ridge of cholesterol fluxes. An inverse correlation was found in the two parameters, as shown in Figure 3, supporting the above model. However, the correlation seems not to be so decisive. The inhibitions were superior when NPC1L1 activity was impaired. In contrast, FNS excretion was superior when LXR was agonized or ABCG5/G8 was overexpressed (Figure 3, see the boxes in the dashed lines). These findings indicate that other factors are involved in the balancing the activities. For example, increased cholesterol synthesis with atorvastatin treatment augmented FNS excretion without reducing cholesterol absorption [127]. 

Although there may be limitations in the model we propose, it is clear that the BBM provides the location for balancing the fluxes. Cholesterol absorption inhibition and TICE have been investigated independently as therapeutic targets of hypercholesterolemia. Because the two phenomena are mechanistically interrelated, they should be considered together when targeting the small intestine for prevention or treatment.
